# Progress in Nonaqueous Molecular Uranium Chemistry: Where to Next?

**DOI:** 10.1021/acs.inorgchem.3c04533

**Published:** 2024-05-13

**Authors:** Stephen T. Liddle

**Affiliations:** Department of Chemistry and Centre for Radiochemistry Research, The University of Manchester, Oxford Road, Manchester M13 9PL, U.K.

## Abstract

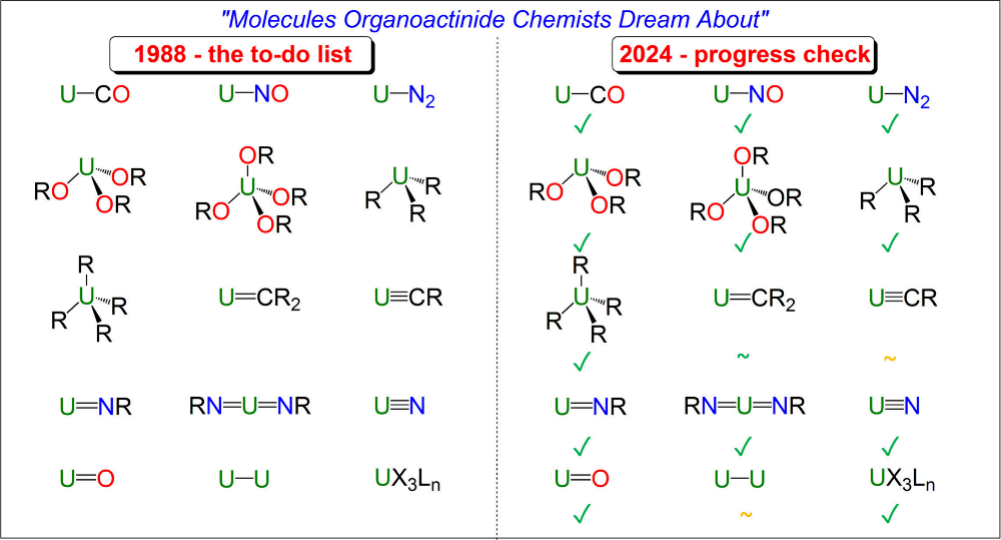

There is long-standing interest in nonaqueous uranium chemistry because of fundamental questions about uranium’s variable chemical bonding and the similarities of this pseudo-Group 6 element to its congener d-block elements molybdenum and tungsten. To provide historical context, with reference to a conference presentation slide presented around 1988 that advanced a defining collection of top targets, and the challenge, for synthetic actinide chemistry to realize in isolable complexes under normal experimental conditions, this Viewpoint surveys progress against those targets, including (i) CO and related π-acid ligand complexes, (ii) alkylidenes, carbynes, and carbidos, (iii) imidos and terminal nitrides, (iv) homoleptic polyalkyls, -alkoxides, and -aryloxides, (v) uranium–uranium bonds, and (vi) examples of topics that can be regarded as branching out in parallel from the leading targets. Having summarized advances from the past four decades, opportunities to build on that progress, and hence possible future directions for the field, are highlighted. The wealth and diversity of uranium chemistry that is described emphasizes the importance of ligand–metal complementarity in developing exciting new chemistry that builds our knowledge and understanding of elements in a relativistic regime.

## Abstract 2

There is long-standing interest in nonaqueous uranium chemistry because of fundamental questions about its basic chemistry. With reference to a “to-do” list from around 1988 that advanced a defining collection of top targets, this Viewpoint surveys progress against those targets. Opportunities to build on that progress and hence possible future directions are highlighted. The wealth and diversity of uranium chemistry that is described emphasizes the importance of ligand−metal complementarity.

## Introduction

**Figure 1:**
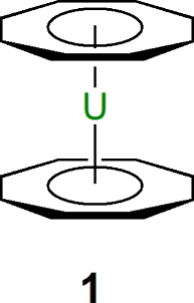
Revolutionary molecule uranocene **1**.^3,4^

Being subject to a rich interplay of relativistic, interelectronic repulsion, spin–orbit coupling, and crystal field effects, the chemistry of actinides is complex and fascinating, and there remains much to learn about these still somewhat enigmatic elements at a basic level.^1^ From a molecular perspective, uranium, in depleted or natural forms, is one of the more intensively investigated actinides. This is not only because of its prominent role in nuclear technologies—with associated extraction, recycling, and cleanup legacy challenges—and relative ease to work with as a weak α-emitter but also because of fundamental questions over the nature of its chemical bonding. With variable oxidation states and a large range of valence orbitals available for hydridization with ligand frontier orbitals, uranium can behave like a covalent transition metal through to being rather ionic like trivalent lanthanides.^2^ Indeed, the fact that uranium was originally classified as a Group 6 transition metal until its rightful place in the 5f actinide series was recognized underlines just how variable the chemical bonding of uranium can be.^1^ Given the need for new knowledge and understanding in nuclear research, for many years the molecular chemistry of uranium was dominated by aqueous studies of the uranyl dication (UO_2_)^2+^.^1,2^ However, seeking to answer the question of how transition-metal-like uranium can be and the role of 5f, 6p, 6d, 7s, and 7p orbitals in its chemical bonding, a debate sparked by the revolutionary molecule uranocene [U(η^8^-C_8_H_8_)_2_] (**1**; Figure 1) from Streitwieser and Raymond,^3,4^ nonaqueous uranium chemistry has flourished over the past four decades.^1,2^ Underpinning all of the advances that have been made in nonaqueous uranium chemistry, and indeed more widely in aqueous studies, is the concept of ligand–metal complementarity because variation of the steric and electronic properties of ancillary ligands is key to enabling and developing new uranium structural motifs, reactivity, and physicochemical properties.

**Figure 2:**
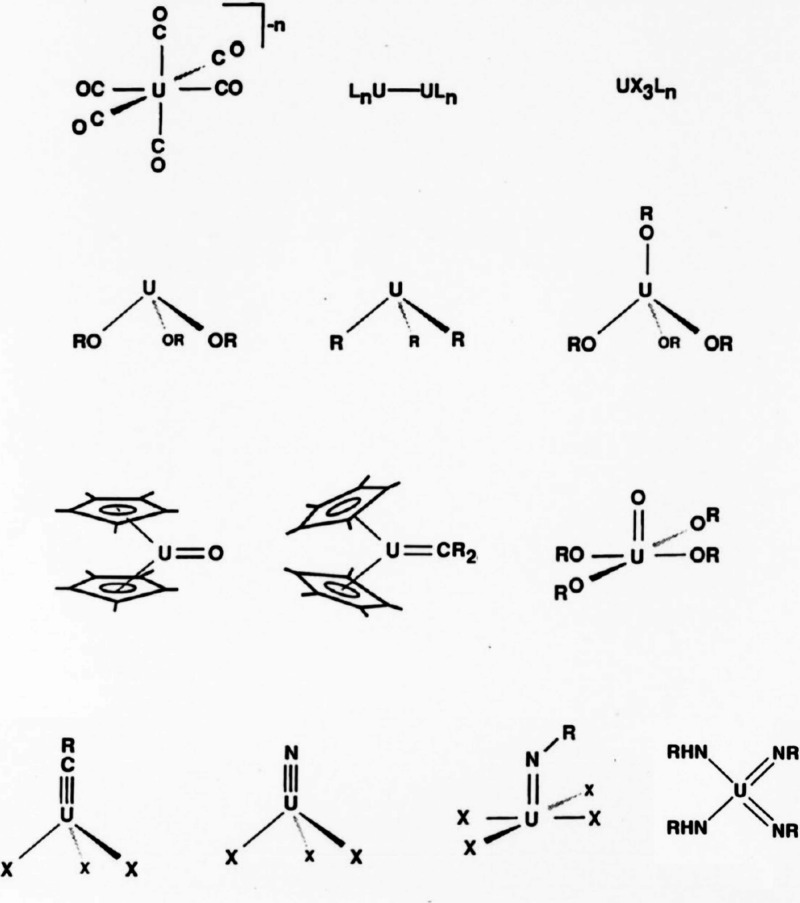
Adapted version of “that slide” on *Molecules Organoactinide Chemists Dream About* from the Los Alamos National Laboratory archive.^5,6^

Reflecting the aforementioned motivation to understand how transition-metal-like uranium is and given an appreciation of uranium’s similarities to molybdenum and tungsten—and hence the likely ability of the former to engage in equivalent bonding motifs to the latter pair—around 1988 there was “that slide” on *Molecules Organoactinide Chemists Dream About*(5) presented by Sattelberger at the Third Chemical Congress of North America (including the 195^th^ American Chemical Society National Meeting) in Toronto that year, an adapted version of which is illustrated in Figure 2.^6^ The slide has since assumed a somewhat legendary status in actinide “folk lore” because it was presented in a conference talk rather than becoming fixed in a journal publication. However, it was an important call-to-arms to the synthetic actinide community to advance the nonaqueous chemistry of uranium in terms of structural linkages that could be isolated under normal experimental conditions. It is intended that, by providing some historical context, viz., Figure 2, and its role in inspiring the progress that followed, the journey and status of the field can be more fully appreciated than by simply presenting advances in isolation.

It is a widely held view that the chemistry of the early actinides lags behind that of the transition metals. However, the astonishing aspect of Figure 2 is just how much was still waiting to be realized ca. 1988 compared to the d block that had undergone major advances in the 1960–1980s. Much has been accomplished in the intervening decades, and so this Viewpoint aims to provide an overview of how the principal themes of Figure 2 developed, and indeed expanded, but will make the occasional detour into motifs or notable analogues with other f elements that assist in contextualizing the area. Hence, the discussion will focus principally on advances directly related to Figure 2 and will then summarize other advances that developed in parallel. The interested reader is referred to several excellent recent reviews and books on the subject, and the cited references herein, for further detailed insight.^1,2,7−21^

## CO and Related π–Acid Ligand Complexes

**Figure 3:**
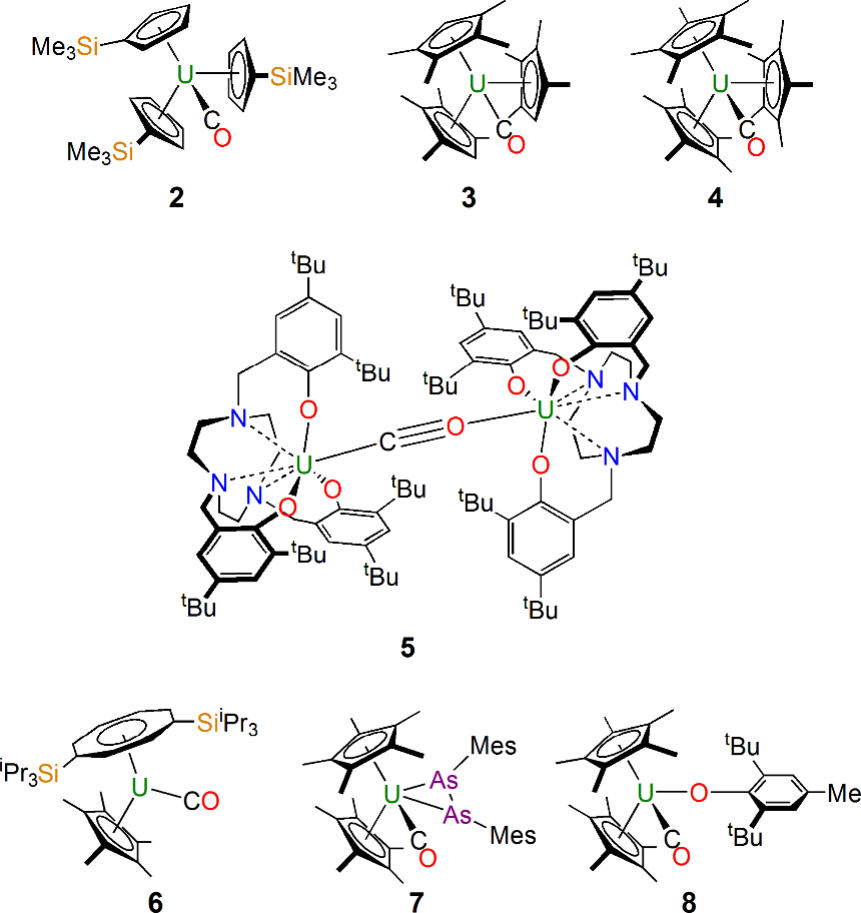
Molecular uranium carbonyl complexes **2**–**8**.^23−29^

There are numerous transition-metal carbonyls; indeed, this is a fundamental class of organometallic complex, so the absence of uranium analogues for many years stood in stark contrast. When Figure 2 was presented, a structurally authenticated uranium carbonyl remained elusive. However, uranium carbonyl had been identified in matrix isolation experiments in 1975 by Sheline and Slater,^22^ and in 1986 spectroscopic evidence by Andersen showed that placing [U(η^5^-C_5_H_4_SiMe_3_)_3_] under an atmosphere of CO produced [U(η^5^-C_5_H_4_SiMe_3_)_3_(CO)] (**2**; Figure 3), but the CO coordination was reversible.^23^ Nevertheless, 1995 marked the first structurally authenticated uranium carbonyl, [U(η^5^-C_5_Me_4_H)_3_(CO)] (**3**; Figure 3),^24^ reported by Parry, Carmona, and Hursthouse. Since then, only a few uranium carbonyl complexes have been reported (Figure 3): [U(η^5^-C_5_Me_5_)_3_(CO)] (**4**) by Evans in 2003;^25^ [{U(tacn[CH_2_C_6_H_2_-2-O-3,5-^t^Bu_2_]_3_)}_2_(μ-CO)] (**5**) by Meyer in 2005;^26^ [U{η^8^-C_8_H_6_-1,4-(Si^i^Pr_3_)_2_}(η^5^-C_5_Me_5_)(CO)] (**6**) by Cloke in 2008;^27^ [U(η^5^-C_5_Me_5_)(As_2_Mes_2_)(CO)] (**7**; Mes = 2,4,6-trimethylphenyl) by Walensky in 2021;^28^ [U(η^5^-C_5_Me_5_)_2_(O-2,6-^t^Bu_2_-4-Me-C_6_H_2_)(CO)] (**8**) by Walensky in 2023.^29^ Evidently, U–CO bonds are not as strong as d-block metal–CO bonds and are hence more difficult to stabilize and isolate.

Interestingly, the IR spectra of **2**–**6** reveal that while the CO stretching frequencies are in the range 1880–1976 cm^–1^, indicating back-bonding into the CO π* orbitals, individual CO stretching frequencies do not correlate with their corresponding Cp–U distances but instead vary with the Cp substituents. In 2009, Eisenstein rationalized this on the basis of U–CO back-bonding from Cp–U bonding molecular orbitals of mainly Cp-ligand character.^30^ Thus, in contrast to the conventional metal-to-ligand back-bonding model for transition-metal π-acid complexes, the back-bonding in tris(cyclopentadienyl)uranium complexes has been classed as ligand-to-ligand back-bonding. Weak ligand-to-ligand back-bonding was also found by Evans and Furche for the cationic thorium complex [Th(η^5^-C_5_Me_5_)_3_(CO)][BPh_4_] reported in 2017,^31^ which, formally, as a 5f^0^6d^0^ metal has no metal-based electrons with which to back-bond. Complex **7** was found to engage in Th–As σ to CO π* back-bonding, and hence that system also engages in ligand-to-ligand back-bonding to stabilize the U–CO linkage.^28^ However, quantum-chemical calculations on **8** suggested that the U–CO back-bonding is from a U 5f/6d hybrid orbital^29^ and hence of metal-to-ligand back-bonding character. The exciting implication is that uranium can switch between ligand-to-ligand and metal-to-ligand back-bonding modes as a function of the ancillary ligands because the only difference between **4** and **8** is the replacement of one pentamethylcyclopentadienyl ligand with an aryloxide. This touches on the variable, responsive bonding nature of uranium, *vide supra*, exemplified by the parallel notion that uranium tends to π-bond to small ligands with mainly 5f character but often bonds to more expansive ligands through δ-bonding with increasing 6d character.^32^

**Figure 4:**
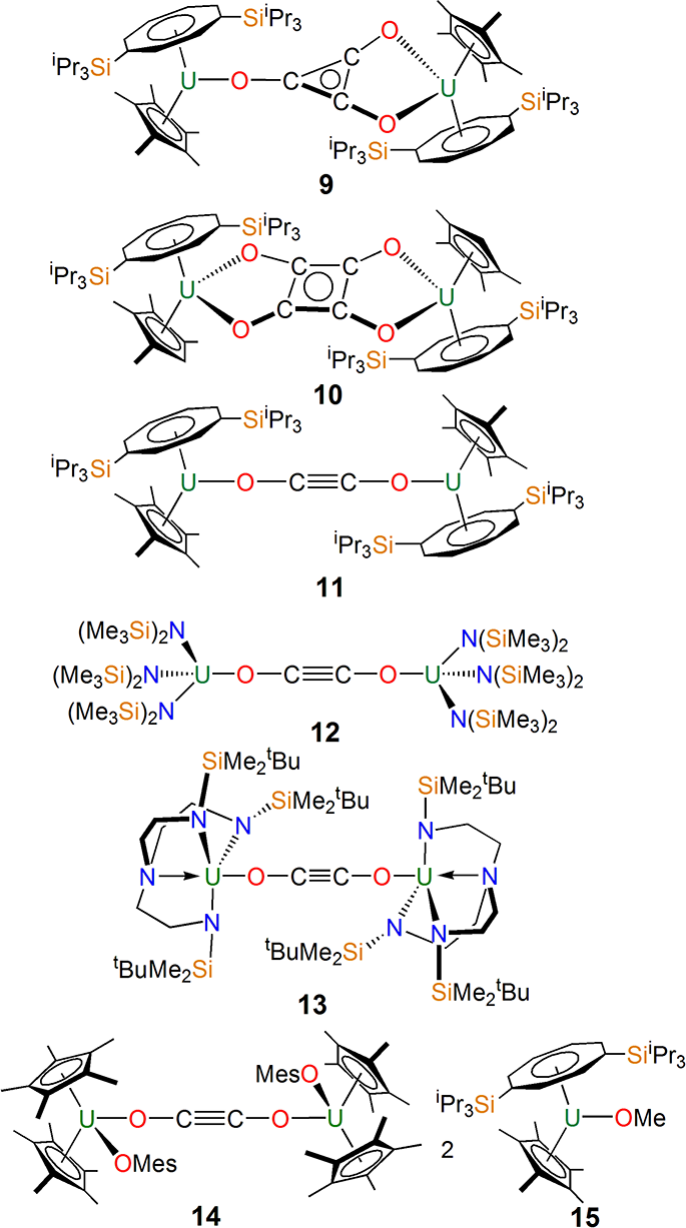
Reductively homologized CO complexes of uranium **9**–**15**.^29,33,35−38^

Complexes **1**–**8** set the scene for reductive homologation of CO at uranium (Figure 4), which contrasts to the more traditional 1,1-migratory insertion chemistry of CO at transition-metal centers. In 2006, Cloke reported the remarkable cyclotrimerization of CO using [U{η^8^-C_8_H_6_-1,4-(Si^i^Pr_3_)_2_}(η^5^-C_5_Me_5_)],^33^ a structurally more sterically demanding analogue of [U(η^8^-C_8_H_8_)(η^5^-C_5_Me_5_)] reported in 1993 by Burns,^34^ to produce the deltate complex [U{η^8^-C_8_H_6_-1,4-(Si^i^Pr_3_)_2_}(η^5^-C_5_Me_5_)]_2_(μ-η^1^:η^2^-C_3_O_3_) (**9**) and then through variation of the Cp substituents or reaction conditions could isolate the cyclotetramerized squarate and dimerized ethynediolate forms of CO in [U{η^8^-C_8_H_6_-1,4-(Si^i^Pr_3_)_2_}(η^5^-C_5_Me_4_H)]_2_(μ-η^2^:η^2^-C_4_O_4_) (**10**)^35^ and [U{η^8^-C_8_H_6_-1,4-(Si^i^Pr_3_)_2_}(η^5^-C_5_Me_5_)]_2_(μ-η^1^:η^1^-C_2_O_2_) (**11**),^27^ in 2006 and 2008, respectively. The formation of ethynediolate at uranium was also accomplished by P. Arnold in 2011 and Liddle in 2012 in [U{N(SiMe_3_)_2_}_3_]_2_(μ-η^1^:η^1^-C_2_O_2_) (**12**)^36^ and [U(Tren^DMBS^)]_2_(μ-η^1^:η^1^-C_2_O_2_) (**13**; Tren^DMBS^ = {N(CH_2_CH_2_NSiMe_2_^t^Bu)_3_}^3–^),^37^ respectively. A synthetic cycle could be closed for the latter where a substituted furanone was liberated,^37^ hinting at a possible catalytic process where uranium meditates the conversion of CO and silyl iodides into a functionalized furnanone. More recently, in 2023 Walensky demonstrated that [U(η^5^-C_5_Me_5_)_2_(O-2,4,6-Me_3_-4-Me-C_6_H_2_)] also reacts with CO to make the ethynediolate complex [U(η^5^-C_5_Me_5_)_2_(O-2,4,6-Me_3_-4-Me-C_6_H_2_)]_2_(μ-η^1^:η^1^-C_2_O_2_) (**14**),^29^ from which a range of complexes featuring further C–C bond-functionalized products could be accessed. A particularly notable result in this arena was the finding by Cloke in 2011 that the complex [U{η^8^-C_8_H_6_-1,4-(Si^i^Pr_3_)_2_}(η^5^-C_5_Me_5_)] reacts with CO and H_2_ to form the methoxide complex [U{η^8^-C_8_H_6_-1,4-(Si^i^Pr_3_)_2_}(η^5^-C_5_Me_5_)(OCH_3_)] (**15**).^38^ The methoxide in **15** could be released as a methanol equivalent in Me_3_SiOMe to, in principle, close a synthetic cycle, and this essentially corresponds to a selective molecular version of Fischer–Tropsch chemistry. Overall, complexes **9**–**15** demonstrate the highly reducing power of low-valent uranium, but thus far this has not gone beyond closed synthetic cycles to true catalysis. This likely reflects unbalanced cycles when factoring in returning uranium to the initial reactive trivalent state.

**Figure 5:**
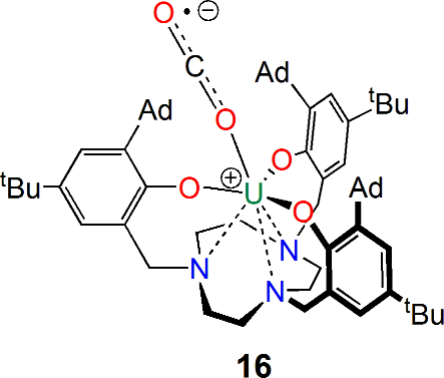
End-on bound uranium–CO_2_ complex **16**.^39^

In parallel to uranium–CO chemistry has been the development of uranium–CO_2_ chemistry. In contrast to the classical 1,2-migratory insertion chemistry of CO_2_, uranium–CO_2_ chemistry took a different turn when Meyer reported the synthesis of the terminal uranium–CO_2_ radical-anion adduct [U{tacn(CH_2_C_6_H_2_-2-O-3-Ad-5-^t^Bu)_3_}(η^1^-OCO)] (**16**; Figure 5) in 2004.^39^ No further reactivity has been reported for that complex, likely because the very steric profile required to stabilize the U–CO_2_ linkage inhibits subsequent reactivity. However, it presented a basis for subsequent studies by Meyer and Mazzanti reporting reductive CO_2_-to-carbonate reactivities including closed synthetic cycles and heteroleptic heavy carbonate analogues.^40−42^

**Figure 6:**
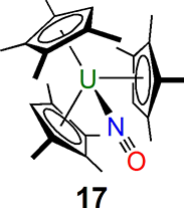
Uranium–NO complex **17**.^44^

Closely related to CO is isoelectronic (NO)^+^, which has an extensive array of coordination chemistry with transition metals. In 1989, Bursten predicted that a [U(η^5^-C_5_H_5_)_3_(NO)] complex would curiously feature a linear U–N–O linkage that could be rationalized as a combination of uranium(IV) Cp_3_U^+^ and not (NO)^+^ but (NO)^−^ fragments, with a further notable prediction of that complex being diamagnetic.^43^ However, experimental validation of those predictions would take 23 years to emerge. In 2012, Evans, Furche, and Long reported the synthesis of [U(η^5^-C_5_Me_4_H)_3_(NO)] (**17**),^44^Figure 6, and it was found to have an essentially linear U–N–O bond angle. Furthermore, quantum-chemical calculations^44^ revealed that the ground state is a diamagnetic singlet, which can be represented as (C_5_Me_4_H)_3_U≡N^+^—O^–^, with a low-lying triplet state corresponding to the U^IV^/(NO)^−^ structure (C_5_Me_4_H)_3_U—N=O, which nicely accounted for the experimentally determined temperature-independent paramagnetism of **17**. Complex **17** remains the sole example of a uranium nitrosyl complex to date.

**Figure 7:**
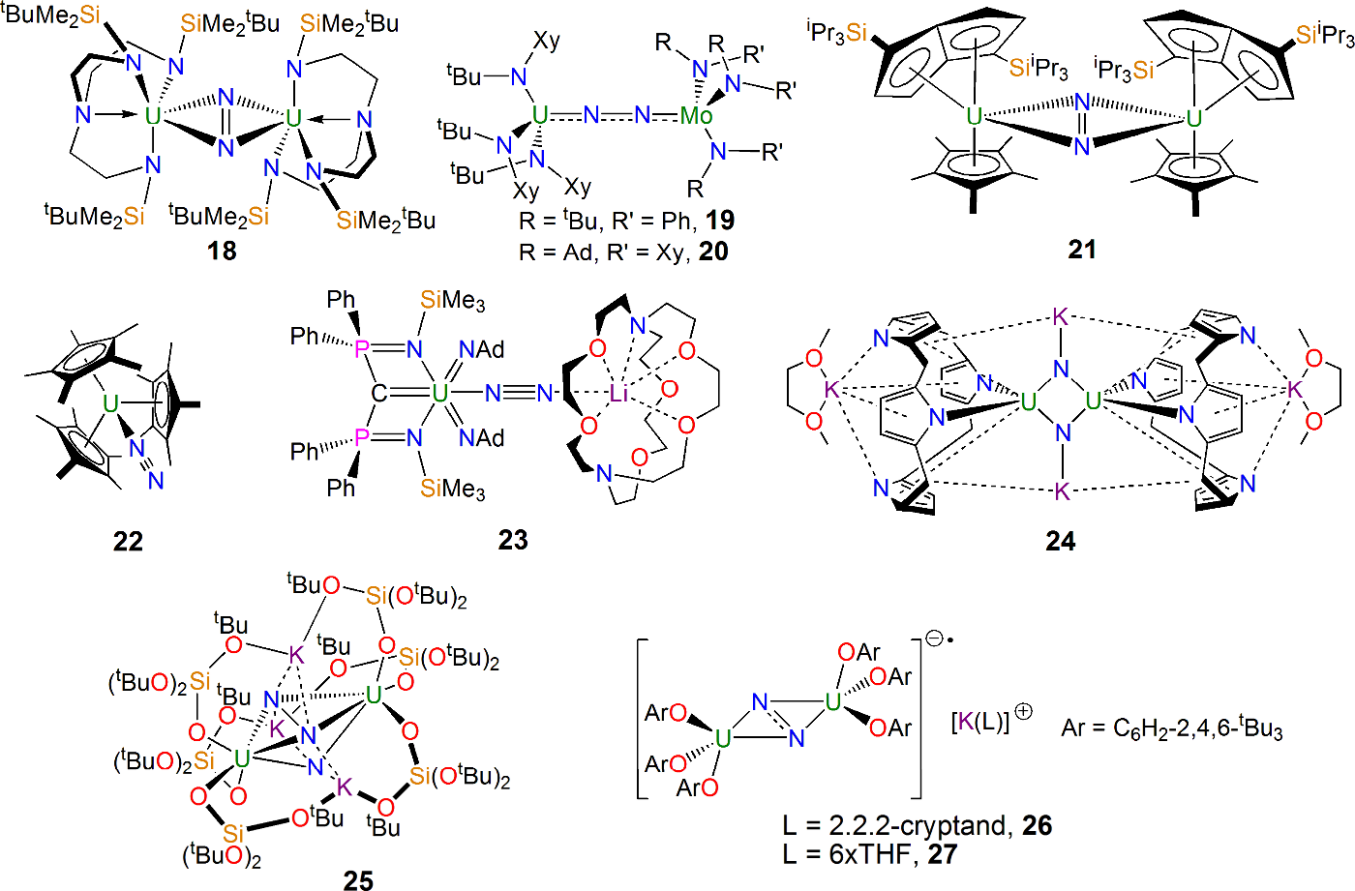
Uranium–N_2_ complexes **18**–**26**.^45,48−54^

With U–CO and U–NO complexes structurally verified and predicted, respectively, by the mid-1990s, attention focused on the essential isoelectronic diatomic N_2_. In 1998, 3 years after **3**, Scott reported the first actinide–N_2_ complex [U(Tren^DMBS^)]_2_(μ-η^2^:η^2^-N_2_) (**18**; Figure 7).^45^ The side-on bridging coordination of N_2_ in that complex was reversible, which led to the initial belief that the uranium ions were trivalent, but it now recognized that N_2_ is reduced to its dianionic form by back-bonding into a π* orbital of N_2_ but reversibly so.

Building on **18**(45) and recognizing the relevance of uranium–N_2_ complexes to Haber–Bosch fixation of N_2_,^46^ in the intervening years to the present day, a range of uranium–N_2_ complexes have been isolated, with most adopting side-on (μ-η^2^:η^2^-N_2_) binding modes that are not reversible.^47^ However, a few of the more unusual end-on or labile side-on-bound derivatives have been reported (Figure 7), including the end-on bridging heterobimetallic complex [{R(R′)N}_3_Mo(μ-η^1^:η^1^-N_2_)U{N(^t^Bu)Xy}_3_] (**19**, R = ^t^Bu, R′ = Ph; **20**, R = adamantyl, R′ = Xy, where Xy = 3,5-Me_2_C_6_H_3_) reported by Cummins in 1998,^48^ [{U(η^8^-C_8_H_4_[Si^i^Pr_3_-1,4]_2_)(η^5^-C_5_Me_5_)}_2_(μ-η^2^:η^2^-N_2_)] (**21**) by Cloke in 2002,^49^ the terminal end-on N_2_ complex [U(η^5^-C_5_Me_5_)_3_(η^1^-N_2_)] (**22**) reported by Evans in 2003,^50^ and the end-on bridging complex [(BIPM^TMS^)U(NAd)_2_(μ-η^1^:η^1^-N_2_)Li(2.2.2-crypt)] (**23**; BIPM^TMS^ = {C(PPh_2_NSiMe_3_)_2_}^2–^) reported by Liddle in 2019.^51^ Complexes **21** and **22** are notable for the facile reversibility of N_2_ coordination, whereas **23** features a high-oxidation-state complex that goes against traditional the donor–acceptor requirements of low-oxidation-state, electron-rich metals.

Other notable achievements in this area (Figure 7) include the splitting of N_2_ into a bis(nitride) in the complex [K(DME)_4_][{K(DME)(Et_8_-calix[4]tetrapyrrole)U}_2_(μ-NK)_2_] (**24**) by Gambarotta in 2002,^52^ hydrogenation to afford ammonia by [{U(OSi[O^t^Bu]_3_)}_2_(μ-N)(μ-η^2^:η^2^-N_2_)K_3_] (**25**) by Mazzanti in 2017,^53^ and recently the formation of N_2_^3–^ at uranium in [K(L)_n_][{U(OC_6_H_2_-2,4,6-^t^Bu_3_)_3_}_2_(μ-η^2^:η^2^-N_2_)] (**26**, L = 2.2.2-cryptand, *n* = 1; **27**, L = THF, *n* = 6) and subsequent N–N cleavage to afford polynitrides by Mazzanti in 2023.^54^ Collectively, these advances highlight the ability of uranium to activate N_2_, confirming the observation that uranium is a highly effective promoter for the formation of NH_3_ from N_2_ and H_2_, as stated in the original Haber–Bosch patent from over a century ago.^46^

## Alkylidenes, Carbynes, and Carbidos

Because the M = CR_2_ (R = H, alkyl, silyl) motif is a fundamental structural class in transition-metal chemistry, there has long been an interest in realizing uranium alkylidenes. However, outside of matrix isolation—where species such as H_2_C=U(X)(Y) (X, Y = F, Cl, Br, I), H_2_C=U(H)X (X = F, Cl, Br), and H_2_C=UH_2_ have been reported by Andrews and Li in the period 2006–2008^55−58^—it is a target that has remained elusive in “pure” M = CR_2_ (R = H, alkyl, silyl) form outside of matrix isolation experiments and so is one of the targets in Figure 2 that remains unmet to this day in isolable molecules made under normal conditions.

**Figure 8:**
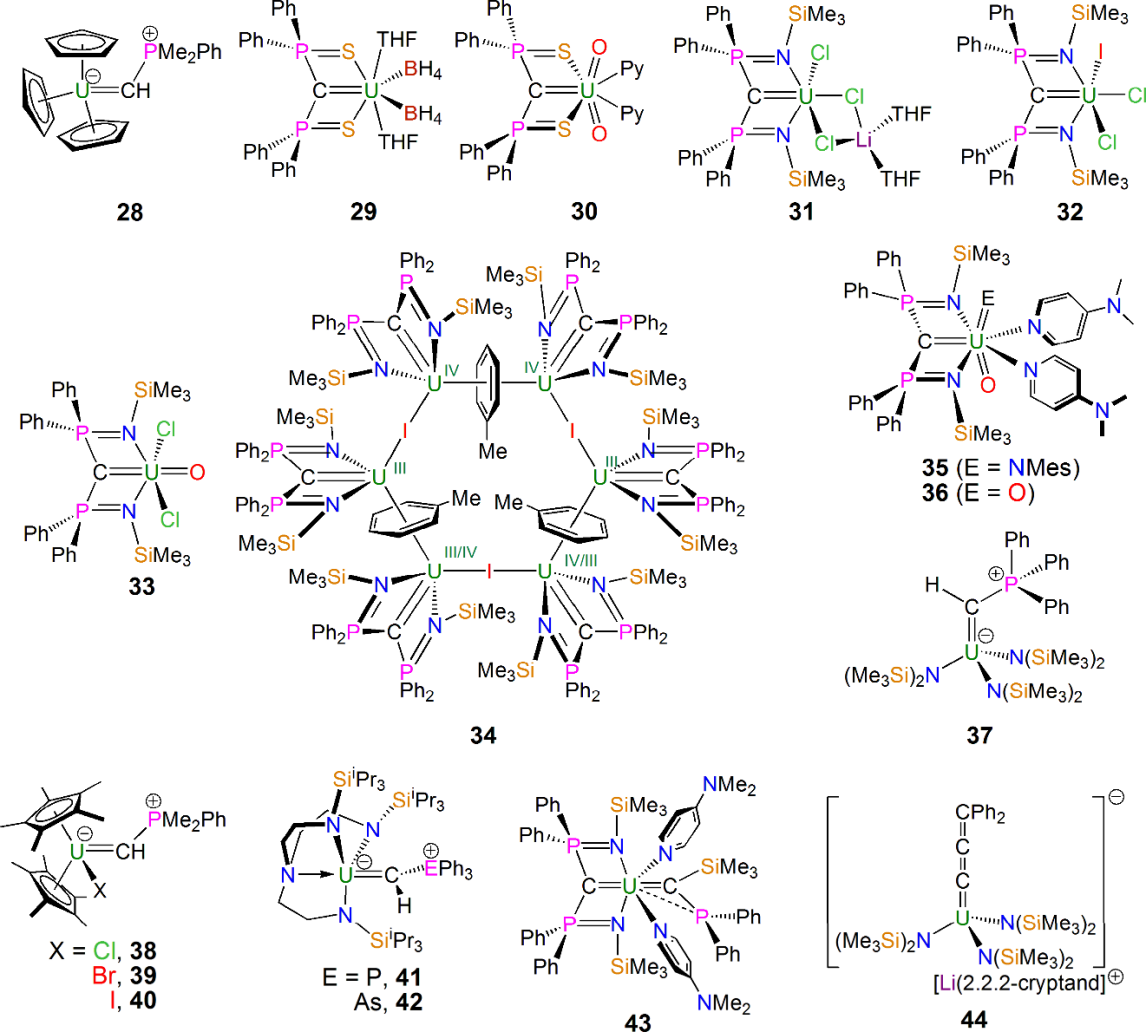
U–C multiple bonds in complexes **28**–**44**.^59−69,71,72^

In 1981, 7 years before Figure 2, Gilje reported the first U=C double bond in [U(η^5^-C_5_H_5_)_3_(CHPMe_2_Ph)] (**28**) by utilizing a phosphonioalkylidene ligand (Figure 8).^59^ The complex undoubtedly contains a U=C multiple bond, albeit polarized, but two competing resonance forms can be drawn [U–C(H)=PMe_3_Ph and U^–^=C(H)-P^+^Me_2_Ph] due to phosphonium-substituent stabilization, which renders the double bond not as clear-cut as that in a “pure” alkylidene. However, a range of reactivity studies were all consistent with UC double-bond character.^12^

The area then became dormant for the best part of three decades before Ephritikhine, Mézailles, and Le Floch revived it in 2009 with the synthesis of U=C double bonds using the diphosphoniomethanediide {C(PPh_2_S)_2_}^2–^ (Figure 8), as exemplified by the uranium(IV) complex [U{C(PPh_2_S)_2_}(BH_4_)_2_(THF)_2_] (**29**),^60^ and then in 2011 the uranyl complex [U(O)_2_{C(PPh_2_S)_2_}(py)_2_] (**30**),^61^ a rare example of a uranyl organometallic. In parallel, with the related diphosphoniomethanediide BIPM^TMS^ (Figure 8), in 2011 and 2012 Liddle reported the uranium(IV), -(V), and -(VI) complexes [U(BIPM^TMS^)(Cl)_3_Li(THF)_2_] (**31**),^62,63^ [U(BIPM^TMS^)(Cl)_2_(I)] (**32**),^63^ and [U(BIPM^TMS^)(O)(Cl)_2_] (**33**),^64^ respectively, allowing comparisons of the U=C bond over three oxidation states of uranium, with the majority of the ligand field conserved. This series was then completed by Liddle and Vlaisavljevich in 2018 with the synthesis of [{U(BIPM^TMS^)}_6_(μ-I)_3_(μ-η^6^:η^6^-C_7_H_8_)_3_] (**34**), which formally contains uranium(III) U=C double bonds^65^ (Figure 8). In 2014, Liddle reported the uranium(VI) derivatives [U(BIPM^TMS^)(O)(NMes)(dmap)_2_] [**35**; dmap = 4-(dimethylamino)pyridine]^66^ and [U(BIPM^TMS^)(O)_2_(dmap)_2_] (**36**),^66^ providing complexes with up to three different multiply bonded ligands at uranium and another rare example of an organouranyl complex (Figure 8). Further prominent examples from the period 2013–2020 of uranium phosphonioalkylidenes (Figure 8) include [U(CHPPh_3_){N(SiMe_3_)_2_}_3_] (**37**) by Hayton and Walensky,^67^ [U(CHPPh_3_)(η^5^-C_5_Me_5_)_2_(X)] (**38**–**40**; X = Cl, Br, I) by Walensky and Maron,^68^ and [U(CHPPh_3_)(Tren^TIPS^)] (**41**; Tren^TIPS^ = {N(CH_2_CH_2_NSi^i^Pr_3_)_3_}^3–^) by Liddle.^69^ The latter provided impetus to prepare the arsonioalkylidene analogue [U(CHAsPh_3_)(Tren^TIPS^)] (**42**),^69^ which was the first arsonioalkylidene complex of any metal and which displays a more well-developed U=C double bond compared to the phosphonioalkylidene analogue, consistent with diminished As versus P stabilization of the alkylidene center. The assertion of the presence of U=C double bonds in these complexes has proven controversial at times, but the weight of reactivity and computational analysis combined with a ^13^C NMR chemical shift anisotropy study in 2024 supporting the Ce=C double-bond formulations in related Ce(IV) complexes^70^ all point to these complexes possessing polarized U=C double bonds.

The years 2018 and then 2021 marked two milestones in U=C double-bond chemistry (Figure 8) with reports of the phosphinosilylalkylidene complexes exemplified by [U{C(PPh_2_)SiMe_3_}(BIPM^TMS^)(dmap)_2_] (**43**) by Liddle^71^ and the allenylidene complex [Li(2.2.2-cryptand)][U(CCCPh_2_){N(SiMe_3_)_2_}_3_] (**44**) by Hayton and Autschbach,^72^ respectively. Both complexes are notable for exhibiting U=C double-bond interactions that depart from the use of pentavalent pnictonium alkylidene stabilization.

Compared to alkylidenes, the corresponding chemistry of uranium carbyne and carbido complexes is sparsely developed. Matrix isolation studies have led the way, with reports of fundamental, elegant species such as CUO, CUO^–^, UC, CUC, UCH, U(CC)_2_, X_3_U≡CH (X = F, Cl, Br), F_2_ClU≡CH, and F_3_U≡CF first being reported around the years 1999–2012 by Andrews, Bursten, and Li.^73−78^ More recently, in recent years (2019–2023), work led by Chen has exploited the unique confinement effects of endohedral fullerenes to isolate a range of carbide compounds, including U(μ-η^1^:η^1^-C)U@C_80_,^79^ U(μ-η^2^:η^2^-C_2_)U@C_78_,^80^ U(μ-η^2^:η^2^-C_2_)U@C_80_,^80^ U(μ_3_-η^1^:η^1^-C)Sc_2_@C_80_,^81^ U(μ-η^1^:η^1^-C)Ce@C_72_,^82^ and U(μ-η^1^:η^1^-C)Ce@C_80_.^82^ Akin to the eventually successful quest for terminal nitrides in isolable molecular species (see below), the prevalence of these species in confined trapping scenarios suggests that, with suitable ancillary ligands, isolable terminal molecular uranium alkylidenes, carbynes, and carbidos under normal experimental conditions should eventually be secured.

## Imidos and Terminal Nitride Complexes

**Figure 9:**
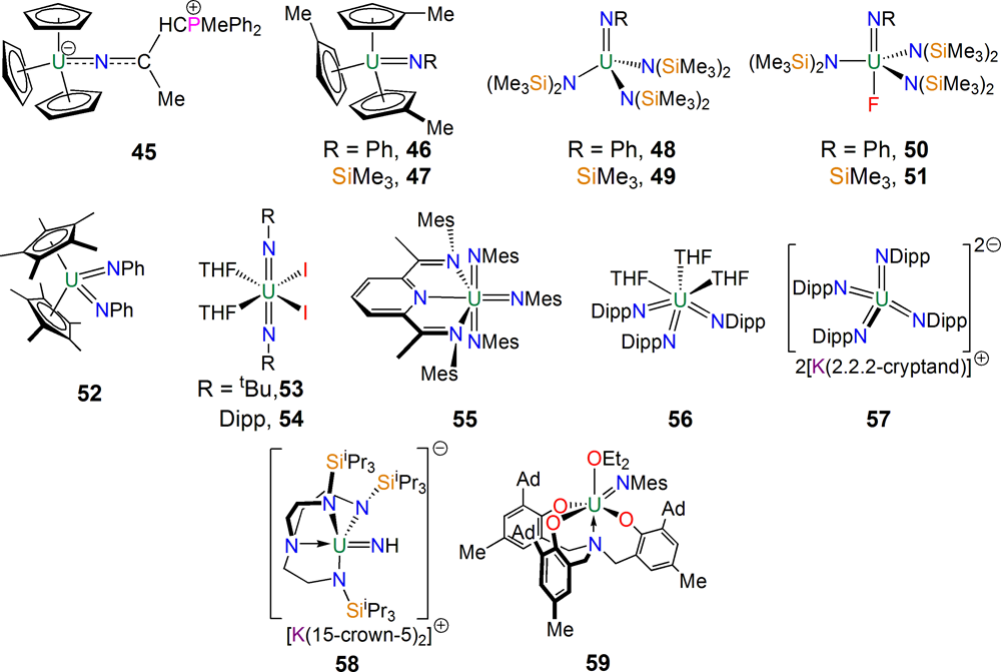
Uranium imido complexes **45**–**59**.^83−96^

By the time the contents of Figure 2 emerged as a presentation slide, uranium mono(imido) complexes had already been realized, and some subsequent key complexes are illustrated in Figure 9.^15^ Initially, in 1984 Gilje isolated the uranium(IV) imido complex [U(η^5^-C_5_H_5_)_3_{NC(Me)C(H)PMePh_2_}] (**45**) from the insertion of CH_3_CN into the U=C bond of the PMePh_2_ analogue of **28**, although this complex is not a “pure” imido linkage.^83^ Soon after, in 1985 Andersen reported two-electron oxidation of [U(η^5^-C_5_H_4_Me)_3_(THF)] by azides to produce the first clear-cut uranium(V) imidos [U(η^5^-C_5_H_4_Me)_3_(NPh)] (**46**) and [U(η^5^-C_5_H_4_Me)_3_(NSiMe_3_)] (**47**),^84^ and the same approach with [U{N(SiMe_3_)_2_}_3_] yielded [U{N(SiMe_3_)_2_}_3_(NPh)] (**48**) and [U{N(SiMe_3_)_2_}_3_(NSiMe_3_)] (**49**) in 1988.^85^ Apart from accessing imido functionalities, these were important reactions because they developed two-electron-oxidation chemistry, in contrast to the reputation that the f block has for one-electron-redox couples. In 1990 Sattelberger reported that **48** and **49** could be oxidized to produce the uranium(VI) imido complexes [U{N(SiMe_3_)_2_}_3_(NPh)(F)] (**50**) and [U{N(SiMe_3_)_2_}_3_(NSiMe_3_)(F)] (**51**),^86^ which were the first uranium(VI) complexes to have multiple bonds to nitrogen.

With mono(imido) uranium complexes established, attention turned to polyimidos, and relatively quickly in 1992 Burns showed that the treatment of [U(η^5^-C_5_Me_5_)_2_(Cl)(Me)] with LiN(H)Ph and Me_2_NCH_2_CH_2_NMe_2_(tmeda) (tmeda = tetramethylethylenediamine) afforded [U(η^5^-C_5_Me_5_)_2_(μ-NPh)(μ-Cl)Li(tmeda)], which was oxidized by N_3_Ph to afford the first uranium bis(imido) complex [U(η^5^-C_5_Me_5_)_2_(NPh)_2_] (**52**),^87^ which was also the first organouranium(VI) complex. Notably, due to the presence of the two Cp* rings, the N–U–N linkage is bent [98.7(4)°], raising interesting questions about its relationship to uranyl and, in particular, the still yet to be routinely isolated *cis*-uranyl. As an aside, noting that **52** was prepared by a two-electron oxidation, in 1993 Burns also found that the oxidation of [U(η^5^-C_5_Me_5_)_2_(ODipp)] (Dipp = 2,6-diisopropylphenyl) and [U(η^5^-C_5_Me_5_)_2_(NDipp)] with pyridine *N*-oxide afforded the first uranium(V) and -(VI) complexes to contain mono(oxo) linkages, namely, [U(η^5^-C_5_Me_5_)_2_(O)(ODipp)] and [U(η^5^-C_5_Me_5_)_2_(O)(NDipp)].^88^ Again, this demonstrated that the uranium(III/V) two-electron-redox couple is a powerful vehicle for installing multiply bonded ligands at uranium.

Complex **52** remained the only class of uranium bis(imido) complexes for 13 years (the *N*-adamantyl version of **52** was reported in 1998)^89^ until in 2005–2006 Boncella reported the synthesis of linear uranium bis(imido)uranyl analogues.^90,91^ Oxidation of uranium metal or [U(I)_3_(THF)_4_] with I_2_ in the presence of amines produced alkyl and arylbis(imido) complexes of the form [U(NR)_2_(I)_2_(THF)_2_] (**53**, R = ^t^Bu; **54**, R = Dipp) with the elimination of ammonium iodide salts. The linear formulation of these bis(imido) complexes suggests that an inverse trans influence operates as it does in isoelectronic uranyl. A tris(imido)uranium complex, isoelectronic to UO_3_, was introduced by Bart in 2014.^92^ The complex *mer*-[U{C_5_H_3_N-2,6-(C[Me]NMes)_2_}(NMes)_3_] (**55**) was obtained by the reaction of a highly reduced, i.e., noninnocent, pyridylbis(imino)uranium complex with MesN_3_, where the U(NMe)_3_ component is T-shaped. This was followed soon after in 2015 by another tris(imido) by Bart in a reaction that is elegant by virtue of its simplicity, where the reduction of [U(I)_3_(THF)_4_] by KC_8_ in the presence of DippN_3_ produced *fac*-[U(NDipp)_3_(THF)_3_] (**56**).^93^

Remarkably, in 2017 Bart reported that the polyimido motif could be extended to a range of tetrakis(imido)uranate(VI) complexes exemplified by [K(2.2.2-crypt)]_2_[U(NDipp)_4_] (**57**).^94^ Quantum-chemical calculations showed that the significant amount of charge loading resulted in more activated U=NR bonds than in tris(imido) and bis(imido) analogues. It will be interesting to see if a pentakis(imido)uranium complex can be realized, given the range and number of vacant valence orbitals that uranium possesses.

There are now many uranium imido complexes, but two merit specific mention. The first is the parent imido complex [K(15-crown-5)_2_][U(NH)(Tren^TIPS^)] (**58**) reported by Liddle in 2014.^95^ Complex **58** is stable despite lacking any sterically demanding substituent protection at the imido, although the anion formulation of the imido component of **58** evidently plays a role because oxidation of **58** results in disproportionation. The imido complex [U{N(CH_2_C_6_H_2_-2-O-3-Ad-5-Me)_3_}NMes] (**59**) was reported by Meyer in 2012.^96^ Notably, the imido resides trans to one of the aryloxides, where it would be more intuitive to predict the imido residing in the axial site trans to the tertiary amine. This implies the presence of an inverse trans influence in **59**.

The search for terminal uranium nitrides can trace its origins back to 1976, when Green and Reedy identified UN in a frozen argon matrix.^97^ Then, in the period 1993–2016, fundamental species such as NUN, NUO, NUO^+^, F_3_UN, NUN-H, and U_2_N_2_ were variously reported or studied in matrix isolation or as spectroscopic transients by Andrews, Bursten, Gagliardi, Pyykkö, Roos, Schwarz, and Vlaisavljevich,^98−102^ and UN was reported in the C_82_ endohedral fullerene by Chen and Autschbach in 2022.^103^ Nevertheless, when Figure 2 was making its debut in 1988, placing an emphasis on a molecular terminal uranium nitride as a key synthetic target and bonding benchmark, there were no molecular uranium nitrides at all.

**Figure 10:**
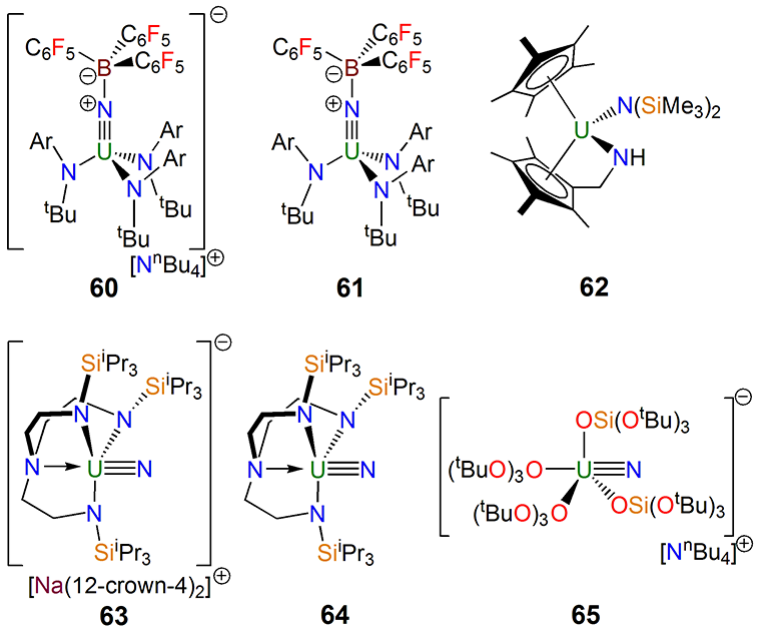
Notable uranium nitride complexes **60**–**65**.^105−108,111^

Several polymetallic nitrides of uranium were reported in the 2000s^21,104^ before (Figure 10) Cummins reported the borane-capped nitride complexes [NBu^n^_4_][U{NB(C_6_F_5_)_3_}{N(^t^Bu)C_6_H_3_-3,5-Me_2_}_3_] (**60**) and [U{NB(C_6_F_5_)_3_}{N(^t^Bu)C_6_H_3_-3,5-Me_2_}_3_] (**61**) in 2009.^105^ Complexes **60** and **61** can alternatively be formulated as imidoborates, but computational analysis reveals significant U≡N triple bonds. In 2010 Kiplinger provided evidence of a transient terminal uranium nitride through isolation of the C–H activated complex [U(η^5^-C_5_Me_4_CH_2_NH)(η^5^-C_5_Me_5_){N(SiMe_3_)_2_}] (**62**) resulting from photolysis of the azide precursor [U(η^5^-C_5_Me_5_)_2_(N_3_){N(SiMe_3_)_2_}].^106^

The terminal uranium nitride was finally reported in 2012 by Liddle in the uranium(V) nitride complex [Na(12-crown-4)_2_][U(N)(Tren^TIPS^)] (**63**),^107^Figure 10, prepared by [U(Tren^TIPS^)]-mediated two-electron azide reduction and subsequent sodium sequestration with 12-crown-4 ether. Success hinged on Tren^TIPS^ providing exactly the right size and shape pocket for the nitride, combined with azide activation but stabilization by the sodium cation and then its gentle subsequent removal. In 2013, the uranium(VI) nitride [U(N)(Tren^TIPS^)] (**64**) was prepared by oxidation of **63**(108) (Figure 10), concluding the search for terminal uranium(VI) nitrides previously restricted to spectroscopic experiments as well as confirming the presence of intermediate nitrides in C–H activation such as **62**–**64**. A range of derivatives of **63** proved to be fertile ground for detailed electronic structure investigations.^109^ Complex **64** was computationally predicted^108^ and experimentally confirmed by ^15^N NMR spectroscopy^110^ to contain a highly covalent U≡N triple bond, and more so than Group 6 terminal nitrides, which is an astonishing result that goes to the heart of one of the original motivations behind Figure 2 to elucidate the bonding relationship of uranium to Group 6 elements like molybdenum and tungsten. Only one other class of terminal uranium nitride has since been reported, where photolysis of [NBu^n^_4_][U(N_3_){OSi(O^t^Bu)_3_}_4_] was reported to produce [NBu^n^_4_][U(N){OSi(O^t^Bu)_3_}_4_] (**65**) by Mazzanti in 2020 (Figure 10).^111^

This area has now expanded to include many examples of astonishing small-molecule activations and structural motifs,^21,104^ with notable examples including hydrogenation of **25** to produce ammonia by Mazzanti in 2017^53,112^ and elegant preparations from UX_5_ (X = Cl, Br) and NH_3_ of bis(nitride) complexes containing the cations [(H_3_N)_8_UNUN(NH_3_)_5_U(NH_3_)_8_]^8+^, [(H_3_N)_8_UNUN(NH_3_)_4_(Br)U(NH_3_)_8_]^7+^, and [(H_3_N)_8_UNUN(NH_3_)_3_(Cl)_2_U(NH_3_)_8_]^6+^ reported by Kraus in 2020.^113^

## Homoleptic Polyalkyl, -alkoxides, and -aryloxides

As a fundamental ligand type in organometallic chemistry, there has always been interest since the 1940s in uranium alkyl complexes particularly because at one stage volatile uranium alkyls were candidates for isotope enrichment work in the Manhattan Project.^114^ In the 1980s, Marks pioneered the study of heteroleptic uranium bis(cyclopentadienyl)alkyls, having reported in 1974 that attempts to prepare tetrakis(alkyl) compounds resulted in decomposition.^115^ Likewise, in 1982 Evans concluded that hydride species formed,^116^ although in 1984 Andersen subsequently found that tetrakis(alkyl) complexes could be stabilized as heteroleptic derivatives by the addition of chelating diphosphine ligands to saturate the coordination sphere of uranium, for example, in [U(CH_2_Ph)_3_(Me)(Me_2_PCH_2_CH_2_PMe_2_)] (**66**).^117^ Thus, Figure 2 focused attention on homoleptic polyalkyl complexes of uranium.

**Figure 11:**
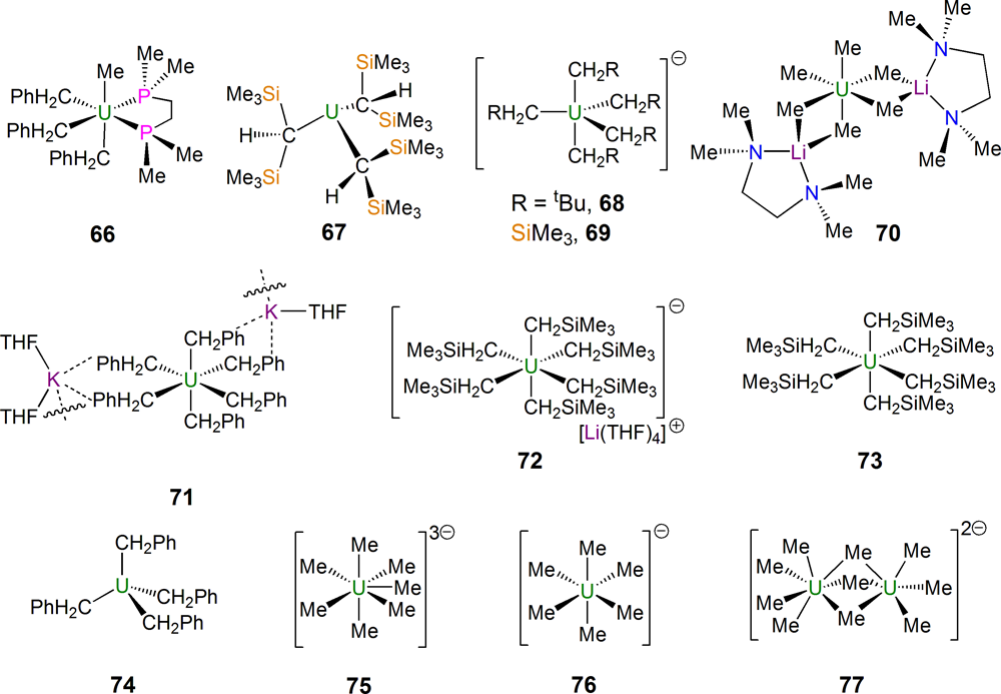
Homoleptic uranium alkyl complexes **66**–**77**. Only the anionic components of **68**, **69**, and **75**–**77** are shown for clarity.^117−123^

As it turned out, a homoleptic polyalkyl was delivered rapidly (Figure 11), and in 1988 Sattelberger reported the first example of a neutral homoleptic uranium alkyl with the synthesis of the tris(alkyl) complex [U{CH(SiMe_3_)_2_}_3_] (**67**).^118^ Like lanthanide analogues, **67** had to be prepared by the reaction of LiCH(SiMe_3_)_2_ with a uranium tris(aryloxide) because the more conventional route of reacting UCl_3_(THF)_*n*_ resulted in formation of the “ate” complex [U{CH(SiMe_3_)_2_}_3_(Cl)Li(THF)_3_].^118^ Complex **67** is isolable because of the sterically demanding alkyls, but it is not coordinatively saturated, so it decomposes in solution, underscoring the inherent reactivity of uranium alkyls.

The year 2009 marked a fresh impetus in the area (Figure 11) when Hayton reported the synthesis of several homoleptic uranium(IV) complexes, specifically separated ion-pair “ate” complexes of the anions [U(CH_2_Bu^t^)_5_]^−^ (**68**) and [U(CH_2_SiMe_3_)_5_]^−^ (**69**) and contact ion triple assemblies of [U(Me)_2_(μ-Me)_4_{μ-Li(tmeda)}_2_] (**70**) and {[K(THF)][K(THF)_2_][U(CH_2_Ph)_6_]}_∞_ (**71**).^119^ Shortly after, in 2011 Hayton went on to report [Li(THF)_4_][U(CH_2_SiMe_3_)_6_] (**72**) and its oxidation to the remarkable hexakis(alkyl) [U(CH_2_SiMe_3_)_6_] (**73**),^120^ although the latter was found to be thermally unstable and decompose above −25 °C. Soon after **72** and **73**, in 2012 Bart reported the synthesis and isolation of [U(η^2^-CH_2_Ph)_4_] (**74**),^121^ where the η^2^-coordination mode of the four benzyls evidently contributes to the stability of this tetrakis(alkyl) complex, and this led to a wide range of [U(η^2^-CH_2_R)_4_] (R = substituted aryls) complexes being reported by Bart in 2015.^122^

As mentioned above, homoleptic polyalkyl complexes of uranium often undergo facile decomposition and can be thermally unstable. This prompted Neidig to undertake low-temperature studies (Figure 11), where compounds were prepared and crystallized at −70 to −80 °C. The resulting range of compounds reported in 2020 underscored the complexity of uranium polyalkyl chemistry because [Li(THF)_4_][U(Me)_4_(μ-Me)_2_{μ-Li(THF)_2_}], [U(Me)(μ-Me)_6_{μ-Li(THF)_2_}{μ_3_-Li(THF)}(μ_3_-Li)] (**75**), [Li(18-crown-6(THF)_2_][U(Me)_6_] (**76**), and [Li(THF)_4_]_2_[Me_4_U(μ-Me)_3_UMe_3_] (**77**), built around hexakis- or septakis(methyl) motifs, could all be isolated under those conditions.^123^

**Figure 12:**
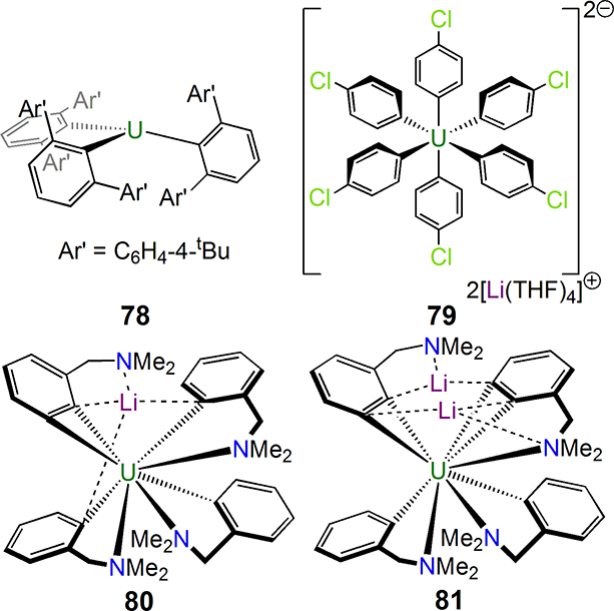
Uranium aryl and benzyne complexes **78**–**81**.^124−127^

The above activity in homoleptic polyalkyluranium chemistry has spurred renewed interest in related homoleptic polyaryluranium chemistry (Figure 12), with notable examples including the uranium(III) tris(terphenyl) complex [U{C_6_H_3_-2,6-(C_6_H_4_-4-^t^Bu)_2_}_3_] (**78**) by J. Arnold in 2016^124^ and uranium(IV) hexakis(aryls) exemplified by [Li(THF)_4_][U(C_6_H_5_)_6_Li(THF)] and [Li(THF)_4_]_2_[U(C_6_H_4_-4-Cl)_6_] (**79**) by Neidig in 2019;^125^ like Neidig’s alkyl work, the latter pair of aryls were synthesized and crystallized at low (−80 °C) temperature. Last, Hayton isolated exceedingly rare examples of uranium benzyne complexes, namely, [U(η^2^-C_6_H_3_-2-CH_2_NMe_2_)(C_6_H_4_-2-CH_2_NMe_2_)_3_Li] (**80**) in 2013^126^ and [U(η^2^-C_6_H_3_-2-CH_2_NMe_2_)_2_(C_6_H_4_-2-CH_2_NMe_2_)_2_Li_2_] (**81**) and the THF-solvate congener [U(η^2^-C_6_H_3_-2-CH_2_NMe_2_)_2_(C_6_H_4_-2-CH_2_NMe_2_)_2_[Li(THF)_2_}(Li)] in 2016.^127^

**Figure 13:**
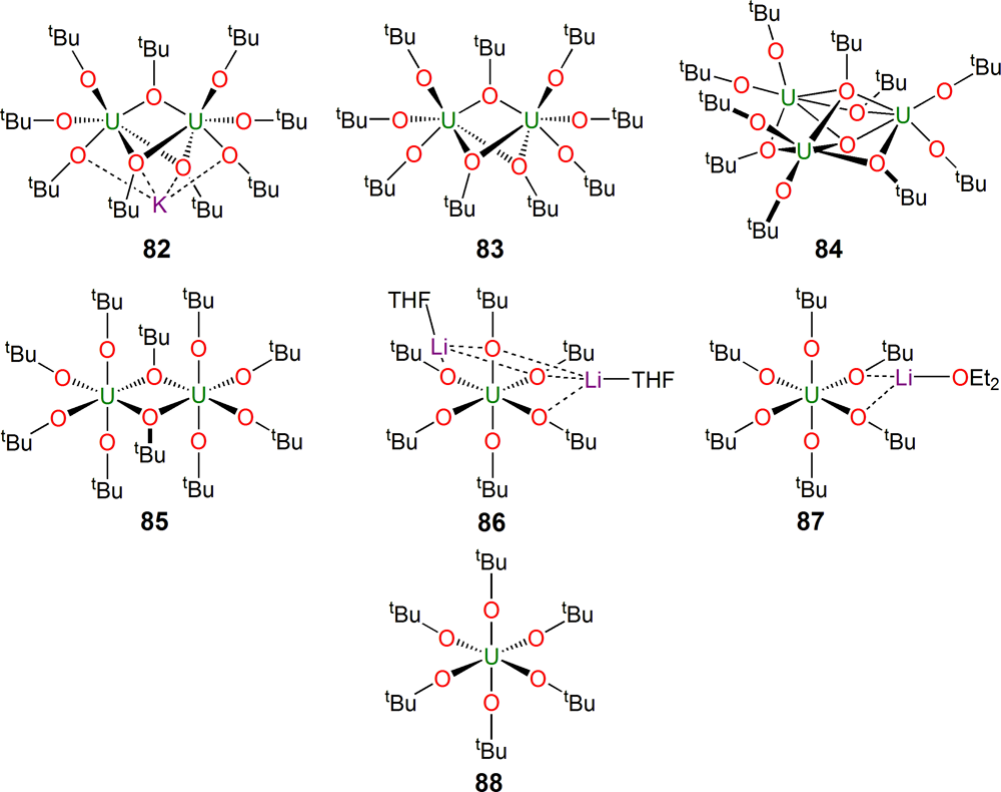
Uranium alkoxide complexes **82**–**88**.^128−132^

Although uranium alkoxides had been known since the 1950s, rather than being straightforward homoleptic formulations, they were often polymetallic aggregates with “ate” character, mixed uranium oxidation states, or were constructed around oxide dianions (Figure 13). Prominent examples include [U_2_(μ-O^t^Bu)_3_(μ_3_-O^t^Bu)_2_(O^t^Bu)_4_K] (**82**), [U_2_(μ-O^t^Bu)_3_(O^t^Bu)_6_] (**83**), and [U_3_(μ_3_-O)(μ_3_-O^t^Bu)(μ-O^t^Bu)_3_(O^t^Bu)_6_] (**84**) reported in 1984 by Cotton,^128−130^ and even the “pure” homoleptic [U_2_(μ-O^t^Bu)_2_(O^t^Bu)_8_] (**85**) reported by Eller in 1983 is dimeric.^131^ Furthermore, aryloxides were relatively scarce, and so Figure 2 sought to prompt an expansion of mononuclear homoleptic polyalkoxides and -aryloxides.

Some of the basic uranium alkoxide chemistry was reinvestigated in 2008 by Hayton,^132^ who found that the tendency of alkoxides to form “ate” complexes could be synthetically exploited. Hence, the preparation of [U(O^t^Bu)_2_(μ-O^t^Bu)_4_{(μ_3_-Li(THF)}_2_] (**86**) was performed, and then stepwise oxidations with iodine first secured [U(O^t^Bu)_4_(μ-O^t^Bu)_2_{μ-Li(OEt_2_)}] (**87**) and then [U(O^t^Bu)_6_] (**86**); it is notable that this chemistry works when utilizing lithium to stabilize the aggregates rather than potassium, which tends to produce clusters such as **82**.^128^ Electrochemical studies suggested significant stabilization of the uranium(VI) ion in **88** compared to the uranium(VI) hexakis(halide) series, which are generally considered to be quite oxidizing.

**Figure 14:**
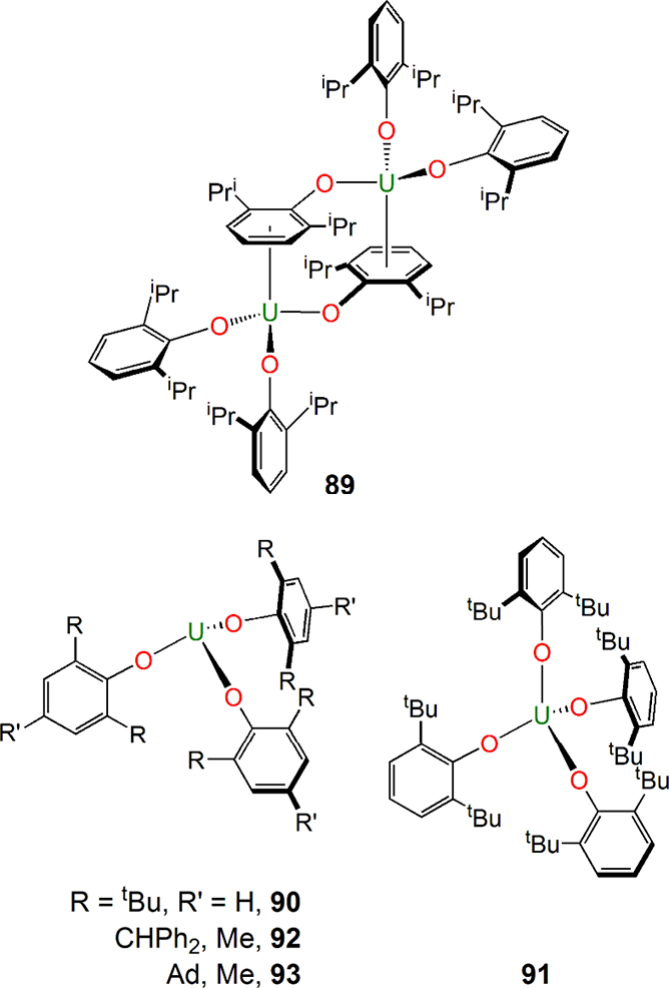
Uranium aryloxide complexes **89**–**93**.^133−136^

Where aryloxides are concerned, there are still relatively few homoleptic variants (Figure 14), with reports by Sattelberger in 1988 of dimeric [{U(μ-η^1^:η^6^-ODipp)(ODipp)_2_}_2_] (**89**)^133^ and the monomers [U(O-C_6_H_3_-2,6-^t^Bu_2_)_3_] (**90**, suggested to be monomeric from IR data in the initial report^133^ but only structurally confirmed as such in 2011 by P. Arnold^134^) and [U(O-C_6_H_3_-2,6-^t^Bu_2_)_4_] (**91**).^135,136^ The more sterically demanding [U{OC_6_H_2_[2,6-CHPh_2_]_2_-4-Me}_3_] (**92**) reported by Meyer and Mindiola^137^ and [U(OC_6_H_2_-2,6-Ad_2_-4-Me)_3_] (**93**) disclosed by Meyer appeared in 2013 and 2016, respectively.^138^ It is worth noting that some homoleptic uranium aryloxides exist but have not been structurally authenticated; however, they have been used to make N_2_^2–^, N_2_^3–^, and CO-coupled ethynediolate derivatives.^54,134^

## U–U Bonds

Given the prevalence of Mo–Mo and W–W bonding in transition-metal chemistry, the absence of U–U bonds led to the latter being a natural target in Figure 2 in 1988. This was not for a lack of attempts to prepare U–U bonds by 1988, where one study by Cotton in 1984^130^ investigating the possibility of accessing U–U bonding supported by alkoxides, given the tendency of alkoxides to support Mo–Mo and W–W bonding, stated that, “While we are not suggesting that on the basis of these two structural results all hope of observing U–U bonds is futile, we do feel that such hopes are rather dim.” Indeed, in 2006 energy decomposition analysis calculations carried out on hypothetical U–U bonds in classical [U_2_X_8_]^2–^ (X = Cl, Br) dianions by Kaltsoyannis^139^ consistently found weak metal–metal bonds. Hence, this suggested that U–U bonds, at least in the [U_2_X_8_]^2–^ formulation, would be unlikely to be formed or be isolable experimentally, in contrast to the large range of heterobimetallic uranium–metal bonds that have been reported.^20^ However, like terminal uranium nitrides, the quest for isolable U–U bonds under normal experimental conditions has been stoked by advances in spectroscopic and trapped-species scenarios.

The U_2_ and OUUO dimers were observed as spectroscopic transients as long ago as 1974 by Khodeev,^140^ and in a theoretical study of actinide dimers by Roos in 2006, there is mention of U_2_ and U_2_^+^ as spectroscopic transients from a private communication from Heaven,^141^ but the nature of the bonding in U_2_ has proven to be a challenge to definitively model due to the relativistic regime.^142,143^ In 1996 and 1997, Andrews showed that HUUH and H_2_UUH_2_ form in cryogenic matrix isolation experiments.^144,145^ It took until 2018 in a report by Chen, Feng, Echegoyen, and Poblet for U_2_ to be formed and isolated in U_2_@C_80_,^146^ although extensive disorder of the U_2_ unit has made analysis of the U_2_ unit challenging. Computational studies suggest a complicated bonding picture that is highly dependent on the U–U distance,^146−148^ but the consensus appears to be that two uranium(III) ions are present with an overall septet spin state but with two ferromagnetic two-center one-electron bonds that correspond to a single bond. Unfortunately, it has not been possible to verify this experimentally due to the lack of magnetic data, which likely reflects the extremely challenging nature of the synthesis and which in itself underscores the achievement of preparing U_2_ at all. The U–U bond in U_2_@C_80_ was described in 2015 by Straka and Foroutan-Nejad as attractive but “unwilling”,^147^ which was debated by Rodríguez-Fortea, Graaf, and Poblet,^148^ but if correct would be in line with prior work suggesting the weak nature of 5f–5f bonding.^130,139^

Interestingly, more recently, in 2021 Th_2_@*I*_*h*_(7)-*C*_80_ was reported by Chen and Poblet^149^ and the trimer [{Th(η^8^-C_8_H_8_)(μ_3_-Cl)_2_}_3_{K(THF)_2_}_2_]_∞_,^150^ accessible under normal experimental conditions and on multigram scale, containing three-center two-electron σ-aromatic bonding,^151^ was reported by Liddle and Kaltsoyannis, also in 2021. These advances in thorium chemistry, together with the matrix isolation and endohedral fullerene advances with uranium, suggest that U–U bonding in a complex made under normal experimental conditions may eventually be realizable.

## Topics That Developed in Parallel to “That Slide”

Figure 2 aimed to capture the spirit of high-value targets to primarily focus efforts on securing. However, of course, it could not envisage every subarea to target or predict what new lines of enquiry those primary endeavors might eventually branch out into, and indeed in many ways, that was also a motivation of Figure 2. This section will briefly summarize other key advances that have branched out in parallel.

One necessary spin-off has been the development of uranium halide starting materials, the importance of which can easily be overlooked when targeting high-value structural motifs, but, of course, the successful isolation of new compounds depends on having suitable starting materials to begin with. There are now many uranium halide starting materials, with UCl_4_ playing a prominent role,^2,15^ but perhaps the one that has had the most obvious sustained impact in terms of uplifting research outputs is that of [U(I)_3_(THF)_4_], reported in publications in 1989 and 1994 by Clark, Sattelberger, and Zwick,^152,153^ for example, already being mentioned above as a key starting point to accessing **53**–**57**.^90,93^

**Figure 15:**
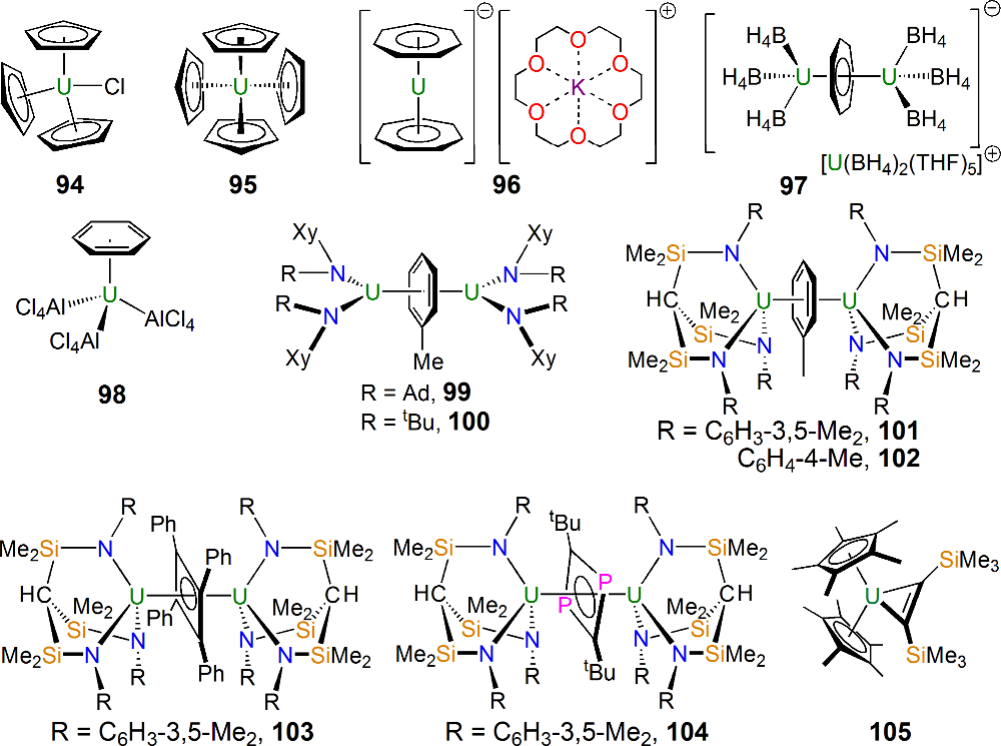
Uranium complexes **94**–**105** with C_*n*_-type ligands (*n* = 2, 4–8).^154−159,161−163^

Many of the linkages in Figure 2 are organometallic, and, of course, organouranium chemistry has a rich heritage spanning back to the 1940s, but definitive compounds began emerging around 1956 and onward, with examples (Figure 15) including [U(η^5^-C_5_H_5_)_3_Cl] (**94**) by Wilkinson in 1956,^154^ [U(η^5^-C_5_H_5_)_4_] (**95**) by Fischer in 1962^155^ [K(18-crown-6)][U(η^7^-C_7_H_7_)_2_] (**96**) in 1995 and [U(BH_4_)_2_(THF)_5_][{U(BH_4_)_3_}_2_(μ-η^7^:η^7^-C_7_H_7_)] (**97**) in 1994 by Ephritikhine,^156,157^ and the aforementioned **1** in 1968/1969.^3,4^ Arene complexes, for example, [U(η^6^-C_6_H_6_)(AlCl_4_)_3_] (**98**) reported by Marconi,^158^ started appearing in the literature around 1971 and onward, although against the backdrop of Figure 2, a notable advance was the report of the inverse-sandwich complexes [{U(N[Xy]R)_2_}_2_(μ-η^6^:η^6^-C_6_H_5_Me)] (R = Ad; **99**, R = ^t^Bu, **100**) in 2000 by Cummins.^159^ There are now numerous inverse-sandwich complexes of uranium, most of which are best regarded as diuranium(III) with arene dianions,^160^ although there are a few notable exceptions of diuranium(V) arene tetraanions, such as [{U(Ts^R^}_2_(μ-η^6^:η^6^C_6_H_5_Me)] [Ts^R^ = {HC(SiMe_2_NR)_3_}^3–^; R = C_6_H_3_-3-5-Me_2_ (Xy), **101**; R = C_6_H_4_-4-Me (Tol), **102**;^161^Figure 15], as unequivocally confirmed by spectroscopic and magnetic studies. The synthetic credentials of **101** and **102** were confirmed by their use as precursors to the first f-element diuranium cyclobutadienyl and diphosphacyclobutadienyl complexes [{U(Ts^Xy^)}_2_(μ-η^5^:η^5^-C_4_Ph_4_)] (**103**) and [{U(Ts^Tol^)}_2_(μ-η^4^:η^4^-C_2_P_2_^t^Bu_2_)] (**104**) (Figure 15) reported by Liddle in 2013.^162^ Continuing the small-ring theme, Walter, Ding, and Zi reported uranium metallacyclopropene complexes such as [U(η^5^-C_5_Me_5_)_2_(η^2^-Me_3_SiCCSiMe_3_)] (**105**).^163^ A recurring theme of **94**–**105** is significant 5f-orbital contributions to the bonding, including π- and δ-bonding motifs, again emphasizing once again how uranium, like transition metals, can engage in different bonding depending on the nature of the coordinated ligands.

**Figure 16:**
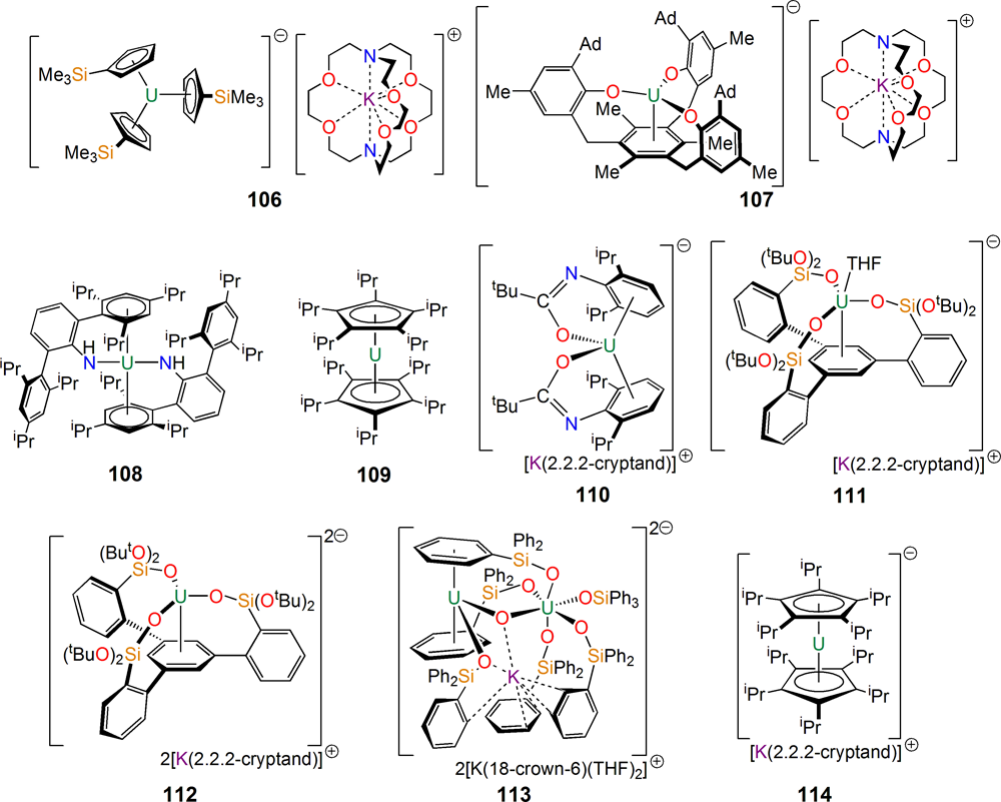
Reduced uranium(II) and (I) complexes **106**–**114**.^164−171^

Although not directly a result of inverse sandwich arene complexes, the oxidation state ambiguity of inverse sandwich arene complexes certainly prompted thoughts of uranium complexes with oxidation states below 3+. Thus, related to inverse sandwich uranium arene complexes, the chemistry of uranium in 2+ and 1+ oxidation states was developed (Figure 16). The first isolable uranium(II) complex was [K(2.2.2-cryptand)][U(η^5^-C_5_H_4_SiMe_3_)_3_] (**106**) reported by Evans in 2013,^164^ and then in 2014, Meyer reported [K(2.2.2-crypt)][U{η^6^-C_6_Me_3_(CH_2_C_6_H_2_-2-O-3-Ad-5-Me)_3_}] (**107**).^165^ These compounds were both important in terms of formally containing uranium(II) but also because the former was found to be 5f^3^6d^1^ and the latter 5f^4^6d^0^. That is a clear demonstration of how the ligand field at uranium can determine the electronic ground-state structure, which is very transition-metal-like behavior. This subarea has expanded significantly, with several ligand classes supporting uranium(II), including the terphenylamide [U{N(H)C_6_H_3_-2,6-[C_6_H_2_-2,4,6-^i^Pr_3_]_2_}_2_] (**108**) by Odom Boncella, and Shores in 2018,^166^ the parallel metallocene [U(η^5^-C_5_^i^Pr_5_)_2_] (**109**) by Layfield in 2020,^167^ the amidate [K(2.2.2-cryptand)][U{OC(^t^Bu)N-η^6^-Dipp}_2_] (**110**) by J. Arnold in 2021,^168^ and the arene-tris(siloxide) [K(2.2.2-cryptand)][U{C_6_H_3_-1,3,5-(C_6_H_4_Si[O^t^Bu]_2_O)_3_}(THF)] (**111**) by Mazzanti in 2023.^169^ Several uranium(I) synthons have now been isolated, including **110** by J. Arnold in 2021,^168^ the arene-tris(siloxide) [K(2.2.2-cryptand)]_2_[U{C_6_H_3_-1,3,5-(C_6_H_4_Si[O^t^Bu]_2_O)_3_}] (**112**) by Mazzanti in 2023,^169^ and [K(THF)_2_(18-crown-6)]_2_[K{(Ph_3_SiO)U}(μ-O)(μ-κ^2^:η^6^-Ph,O-PhSiPh_2_O)(μ-κ^2^:η^4^-Ph,O-PhSiPh_2_O){U-(Ph_3_SiO)_3_}] (**113**) also by Mazzanti in 2023.^170^ Uranium(I) has been identified in disordered [K(2.2.2-cryptand)][U(η^5^-C_5_^i^Pr_5_)_2_] (**114**) by Layfield.^171^ These results have paralleled advances isolating thorium(III) and, remarkably, thorium(II) in molecular tris(cyclopentadienyl) complexes.^172−174^ This has even been extended to include neptunium(II)^175^ and plutonium(II),^176^ showing the impact that studying uranium can have on neighboring actinide elements.

**Figure 17:**
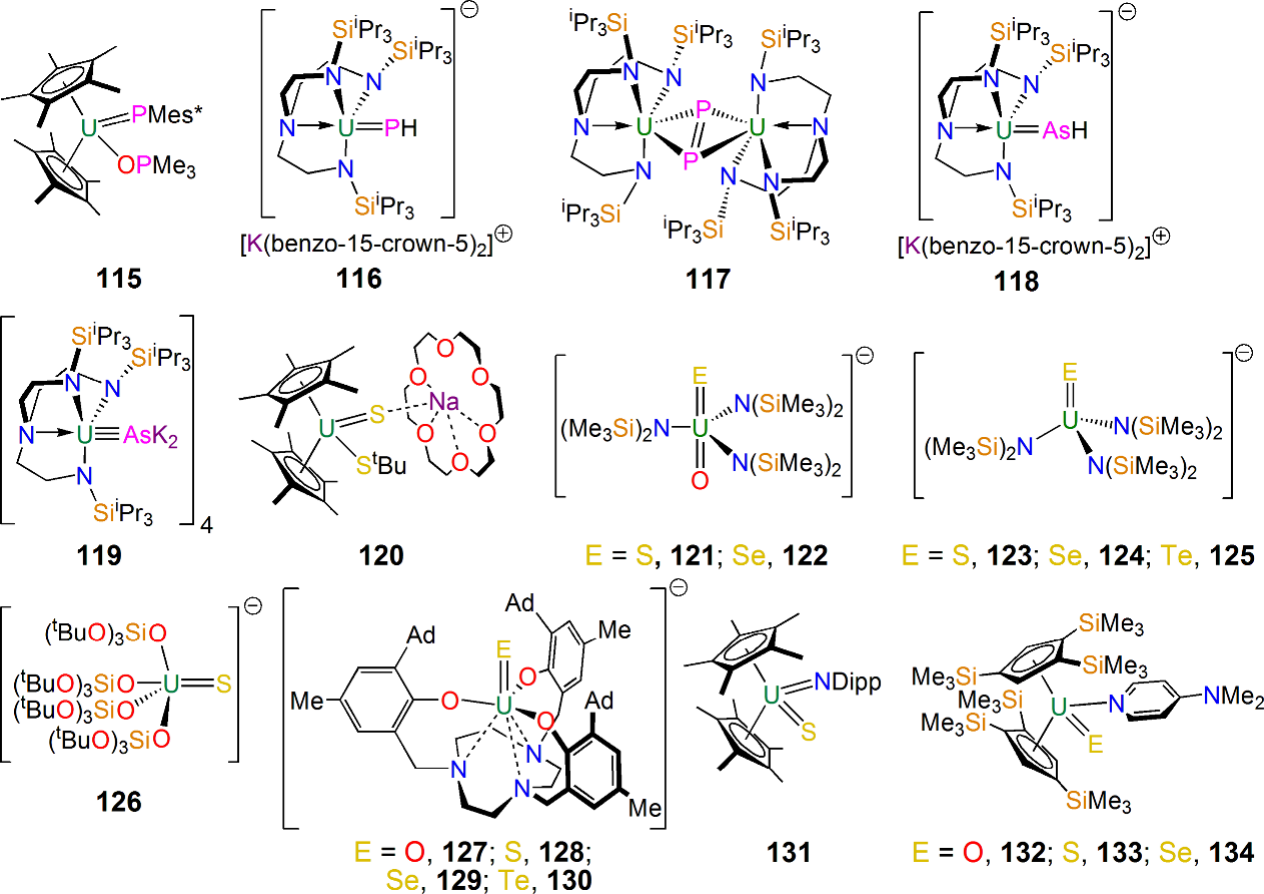
Selected examples of heavier Group 15 and 16 multiple bonds to uranium including **115**–**134**.^177−184^ Mes* = 2,4,6-^t^Bu_3_C_6_H_2_. Dipp = 2,6-^i^Pr_2_C_6_H_2_. Cation components of **121**−**130** are omitted for clarity.

As indicated above, there are now many amides,^15^ imidos,^16^ nitrides,^21,104^ and oxos,^9−12,15^ so attention naturally turned to developing to accessing multiply bonded heavier group 15 and 16 derivatives of uranium by way of phosphinidene, phosphido, diphosphorus, arsinidene, arsenido, sulfido, selenido, tellurido, and Zintl cluster complexes.^177−183^ The result is that there is now a significant range of U=PR (R = H, aryl), U=P(R)K, U=P=U, U–P(H)–U, U(P_2_)U, U(P_3_)U, U=AsR, U=As(R)K, U=As=U, U≡AsK_2_, U(As_2_)U, U(As_2_H_2_)U, U=S, U=Se, and U=Te bonds reported with a range of supporting ligands. A selection of representative complexes reported by Burns, Liddle, Ephritikhine, Hayton, Mazzanti, Meyer, Kiplinger, and Walter can be found in Figure 17 (**115**–**134**), and the reader is directed to recent reviews^177,178^ and subsequent publications.^179−184^ Overall, the range of heavier Group 15 and 16 derivatives emphasizes how multiple bond linkages more often associated with the d block can be stabilized and isolated at uranium through appropriate synthetic approaches coupled to ligand–metal complementarity.

In addition to all of the above fascinating chemistry, the long-known uranyl dication has continued to produce new chemistry time and time again. Although the uranyl dication is often referred to as inert, Clark showed in 1999 that, under highly alkaline conditions, oxo–ligand exchange can occur in uranyl hydroxides.^185^ In the years that followed, uranyl activation developed into two distinct but interrelated areas, that of pentavalent uranyl and its disproportionation chemistry, and functionalization of uranyl producing O-element bonds from the “yl” oxos, which often involved reduction and hence pentavalent uranyl-type intermediates.^186−193^ Through a range of silylation and borane-silylation chemistry, activation of uranyl and reduction to uranium(IV) species is now well-established, which when taken together with the facile oxo exchange by Clark renders the classical textbook description of the inert nature of the uranyl dication, except for in acidic media, somewhat in need of revision. Another textbook description of uranyl is that it is rigorously linear, but several studies have now reported uranyl O–U–O angles of ∼162–168°.^66,194,195^ Furthermore, *cis*-uranyl was proposed by Meyer in 2023 as a credible reaction intermediate,^196^ suggesting that with suitable trapping a *cis*-uranyl may be within reach, which would also contribute to a need to rewrite textbook descriptions of uranyl. Last, there is continued interest in the extraction of uranyl, with a recent highlight being redox-switchable carboranes for uranium capture and release reported by Ménard and Hayton in 2020.^197,198^ Again, all of these advances rely on ligand–metal complementarity to be successful.

Earlier, this Viewpoint touched on small-molecule activation and catalysis by uranium, mainly with CO, CO_2_, and N_2_, but uranium has a rich chemistry in this area with a range of small molecules and substrates,^8,12,19^ even, in nonaqueous media, remarkably including water splitting reported by Meyer.^199,200^ This is just one subarea of several novel physicochemical properties that uranium exhibits by virtue of its position in the Periodic Table, with others including studies encompassing single-molecule magnetism,^201^ the inverse trans influence,^202^ 6p-orbital pushing from below,^203^ sterically induced reduction chemistry,^204^ and even noble gas adducts under matrix isolation conditions.^205^

With such a rich range of new molecular complexes to study and with characterization techniques and methods becoming ever more capable and widely available, there has been growing interest in probing the covalency of uranium complexes; after all, this goes to the very heart of one of the prime motivations for pursuing molecular nonaqueous uranium chemistry, and methodological advances mean that studies that would have been unimaginable in 1988 are now verging on becoming relatively routine. From 2009 and onward, ligand K-edge X-ray absorption spectroscopy (XAS) has enabled uranium-ligand covalency to be probed from the perspective of the ligand,^206−212^ and increasingly resonant inelastic X-ray spectroscopy (RIXS)^213−215^ is providing a complementary perspective from the metal side. However, given that covalency can be understood and defined^216,217^ as the spatial overlap of parent atomic orbitals or near-energy matching of parent atomic orbitals, or simply the net amalgamated result of both, precisely what XAS and RIXS data are reporting is an interesting debate.^218^ Pulsed electron paramagnetic resonance spectroscopy has now been used to probe unpaired spin density,^219^ although again how that relates exactly to describing covalency is an interesting question. Optical spectroscopy has been used to quantify 5f-orbital covalency and can be the basis of a quite detailed dissection of uranium bonding, but so far this has been limited to probing only the 5f-orbital contributions.^110,220−222^ Last, NMR spectroscopy has emerged as a powerful way to probe the covalency of molecular actinide–ligand linkages, where a detailed interrogation of the shielding parameters can quantify the bonding. However, this approach is currently restricted to diamagnetic complexes and so has focused on uranium(VI) and thorium(IV) complexes.^111,223−231^

## Conclusions and Outlook

**Figure 18:**
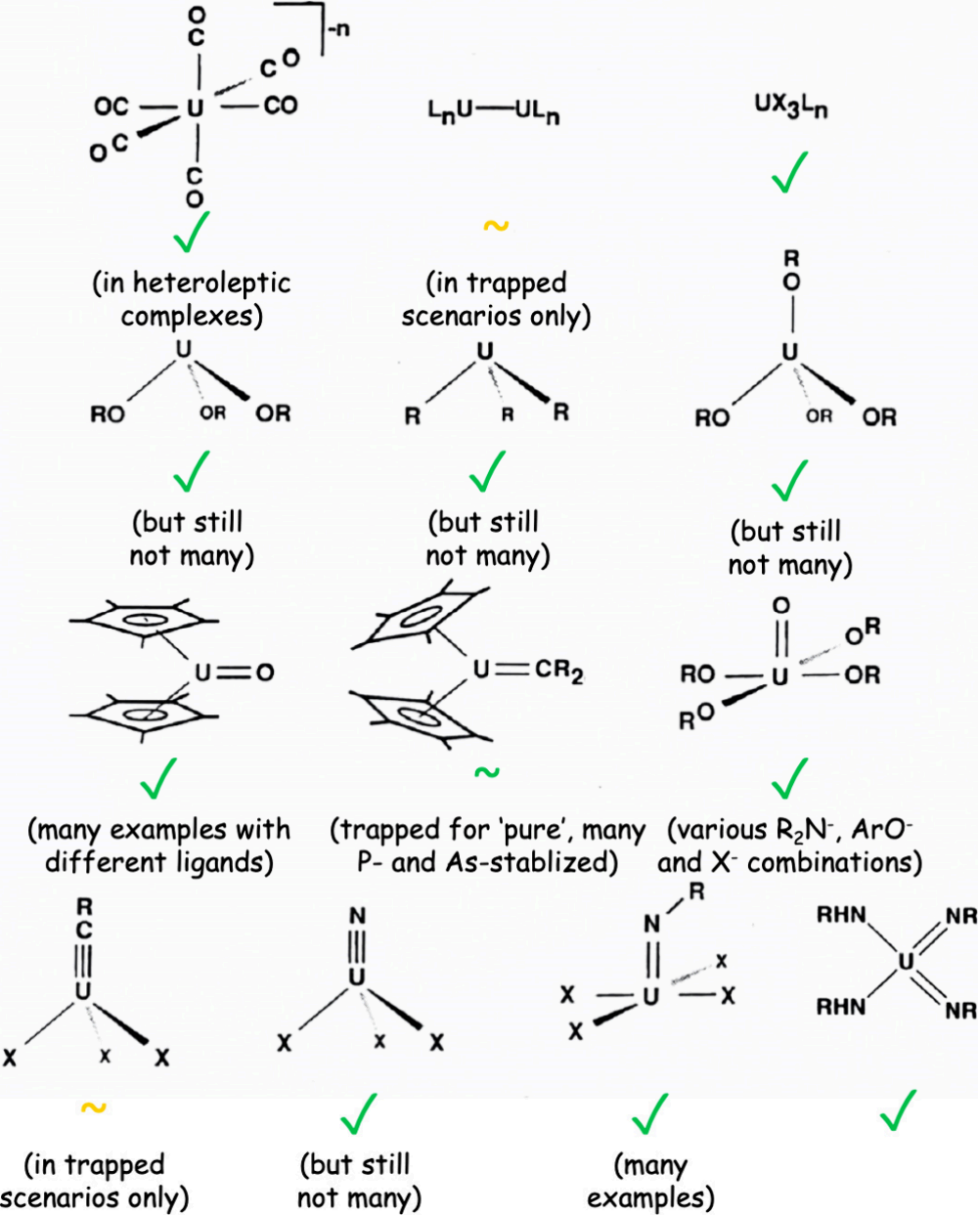
Updated version of Figure 2, the result of ∼36 years of progress.

Some 36 years after the vision of Figure 2 first emerged, this Viewpoint has sought to highlight the broad range of resulting advances that have directly, or in parallel, been delivered. An updated version of Figure 2 is presented in Figure 18, showing that most of the major targets have been secured or have close approximations. Many advances have resulted, and in particular an ever better understanding of chemical bonding in a relativistic regime has been developed, and the redox chemistry of uranium has proven to be exploitable in numerous scenarios to secure new bonding motifs, reactivity, and physical properties. Perhaps one of the most important advances is the knowledge that even targets likely initially thought to be more aspirational than actually achievable were eventually secured—persistence is the victor.

What started as a presentation slide now requires this Viewpoint to barely scratch the surface of all of the advances that have occurred. That underscores just how much has been achieved in the intervening four decades, and those advances have undoubtedly prompted the community to reevaluate the nature of actinides. This naturally leads to the question, “Where to next?” While not claiming to be a definitive and exclusive list, the following emerge as obvious areas of focus:

- A “pure” alkylidene linkage of the form M=CR_2_ (R = H, alkyl, silyl) is yet to be secured in an isolable molecular actinide complex under normal experimental conditions.
- Actinide carbyne and carbido complexes, in particular terminal variants, are yet to be secured in an isolable molecular actinide complex under normal experimental conditions.
- Heavier Group 14 and 15 element bonding to uranium requires further development.
- U–U bonding in an isolable molecular complex under normal experimental conditions is yet to be secured.
- A clear-cut *cis*-uranyl in an isolable molecular complex under normal experimental conditions is yet to be secured.
- The above all emphasize a need to develop the molecular chemistry of transuranium elements. Noting recent reports on a neptunium(V) bis(imido) in 2015,^232^ a neptunium(V) mono(oxo) in 2022,^233^ and neptunium(III) and plutonium(III) diphosphonioalkylidenes in 2022 and 2024,^234,235^ respectively, and early reports of alkyls and alkoxides that lack definitive structural authentication, many of the bonding motifs from Figure 2 that have been delivered with uranium demand realization in transuranium chemistry. This applies to thorium as well, although to a lesser extent given recent advances in its chemistry. It is also worth noting that protactinium chemistry is arguably the “sleeping beauty” of the actinides whose development is long overdue.
- All of the areas listed under parallel topics above would also certainly benefit from being translated to transuranium analogues in order to truly build a rigorous picture of actinide periodic trends.

The prior discussion above is not exhaustive by any means but aims to provide context, highlight what has been done and why, and perhaps provide inspiration to focus attention onto the possible opportunities and directions of future travel that researchers in the area might pursue. Finally, the above also serves as a powerful example of the importance of ligand–metal complementarity in developing exciting new chemistry to build our knowledge and understanding of the f elements, especially in a relativistic regime.
